# Notes on Cases of Necrosis of the Lower Jaw in Children

**Published:** 1894-01-13

**Authors:** Wheelton Hind

**Affiliations:** Assistant Surgeon, North Stafford Infirmary


### Medical Progress and Hospital Clinics.

[*The Editor will be glad to receive offers of co-operation and contributions from members of the profession. All letters should be addressed to* The Editor, The Lodge, Porchester Square, London, W.]

### NOTES ON CASES OF NECROSIS OF THE LOWER JAW IN CHILDREN.

By Wheelton Hind, M.D., B.S., F.R.C.S., Assistant Surgeon, North Stafford Infirmary.

Necrosis of the lower jaw is a rare disease apart from phosphorus or mercurial poisoning, and little is to be found on the subject in text books.

During the years 1890 and 1891 I saw an extraordinary number of cases of this disease at the North Stafford Infirmary, many of which by the courtesy of one of my colleagues came under my own care, while I also had the opportunity of seeing others under the care of my other *confrères*. The history of the disease was as follows: The patients, all children, aged from three to nine years old, and, therefore, in an active state of dentition, were all brought up for a swelling at the angle of the jaw, in all cases but one unilateral. This swelling was hard, non-circumscribed, and on examination of the mouth there was usually a discharge of pus from the gum behind or near the last cut molar. In a few cases there was also an external sinus. A probe passed into the sinuses came down on bare bone. There was comparatively little fœtor, but owing to the infiltration of muscle, or due to the inflammation of its attachment to the jaw, there was some difficulty in opening the mouth, but there was very little pain after the discharge became developed. The little patients exhibited a characteristic appearance with the much-swollen cheek and absence of any constitutional affection, the majority appearing in fairly good health. They came from all parts of the towns of North Staffordshire, belonged to the poorer classes, and were equally divided between the sexes; none came from the country districts, or were over nine years old, and there was no history of any mercurial treatment, or exposure to the fumes of phosphorus. It was very improbable, too, from the absence of other symptoms, that any phosphorus had been accidentally absorbed from sucking lucifer matches, and from the non-fatal character of the disease.

The limit in time of the epidemic is marked. No cases were seen before 1890, and none have been seen now for more than a year, and this fact has led me to think that one great factor in the causation of this affection was a series of wide-spread epidemics of measles which attacked the district in the years 1889—90—91, though I must confess that a history of measles was not obtained in all of the cases, and which disease attacked a part in which active processes of growth were going on; and I think, too, that the limit of age points exclusively to the fact that the growth and eruption of the permanent six-year-old molars played an important part in the ætiology of the disease, and this I consider to have been the determining factor in the causation of necrosis. In several cases I found lying loose or impacted in the necrotic bone the crown of the six-year-old molar, quite separated from any pulp organ, and without any connection with soft material. The crowns were in all cases fully formed, but there were no signs of fangs or roots, and the tooth seemed to occupy the centre of disease, for, as a rule, the necrosis was limited to the alveolar process posterior to the milk molars and the anterior part of the ascending ramus. It, therefore, seemed to me that the probable train of events was—first, an interference with the growth of the first permanent molar, due to the low state of health induced by zymotic disease; and, secondly, death of the tooth, which, acting as a foreign body, set up necrosis in the surrounding jaw.

The treatment I adopted in all cases which came under my care with necrosis already developed was to improve the general health with cod liver oil, syrup of the iodide of iron, and good feeding. Antiseptic mouth washes of boracic acid, chlorate of potash, and myrrh were used until there was evidence of the sequestra being loose, and when such was the case to make an incision between the gum and the cheek posterior to the milk molars, which I also removed if their bony sockets were involved in the disease. Then, with a small pair of bone or dressing forceps, to remove all the dead bone, making a special point of searching for all loose crowns of the permanent teeth which might be in the necrotic cavity. I would remark here that it is improbable that any but the third permanent molar will be found from the age of the patients, the part of the jaw with the second and third molars not being developed in young children.

In the majority of cases the necrosis appeared to be limited to the outer surface of the jaw, chiefly the alveolar portion and the ascending ramus being affected, but in one case (that of a little girl aged three years), where the disease was bilateral, I removed the whole of the ascending ramus and condyle from one side (the left), and a large sequestra from the surface of the bone with the condyle of the right. A great deal of the swelling in these cases is due to the rapid production of new bone round the necrosed portion; and, indeed, this fact is a very important feature in the subsequent process of healing.

I have been struck in all cases with the very rapid reproduction of the jaw, and the absorption of the swelling around, there being, in the cases I have been able to keep under observation, very little resulting deformity. Even in the case where both condyles were removed, the rapidity with which the power of mastication returned was marvellous.

I saw one case in a lad who gave no history of measles or other zymotic illness. Here the trouble did not end till the impacted crown of the six-year-old molar was removed, and it is quite possible that the case does not belong to the same category as the others, and was due to cystic expansion of the jaw owing to an impacted molar. This was one of my earliest cases; I was, I must confess, puzzled over it. The slow growth, age of child, and general health, made me think of sarcoma, and I prepared the parents for the necessity of the removal of half of the lower jaw; but he came under one of my colleagues, who explored, after finding pus welling up into the mouth and removed some dead bone. Subsequently the case came into my hands for another operation, whilst taking charge of my colleague's cases, and as more bone was loose, I operated for its removal; but a third operation becoming necessary, I felt that there must be some cause of irritation remaining, and found the six year molar impacted, which was absent from the denture, lying obliquely in the horizontal ramus of the jaw. This I removed, and a rapid cure resulted.

The history of swelling and the formation of ossifying callus that I have noted differ from the experience of Mr. Salter, who states in his article on Diseases of the Teeth in "Holmes' System": "There is no swelling, and no ossifying callus is formed in the region of the necrosed bone." It was by this observer that the connection of jaw necrosis with the exanthemata was first pointed out; and he says in nine years he saw twenty-three cases. It would have been interesting to note whether they occurred in epidemics or sporadically.

In one case only, and that in private, did I see the first symptoms of the disease. It came on very rapidly soon after an attack of measles, and after the swelling had lasted two days, I made free incision down to bone, along the posterior part of the gum, with the happy result of arresting the disease. I felt a lot of bare bone which was evidently, however, not so far diseased as to go on to necrosis. Judging from this case only the disease would appear to approach more nearly to the acute periostitis of other bones, and it is quite possible that the poison of the exanthem would find a suitable nidus in that tissue as well as in the pulp organ, and if this is the more correct interpretation of the pathology, the treatment of free incision is that most likely to result in the saving of the bone.

As to differential diagnosis. Sarcoma of the jaw is the most likely disease to be mistaken for this disease, but the welling up of pus or formation of abscess will point to necrosis. Cases of dentigerous cysts will of course also closely resemble necrosis with swelling, and as the line of treatment is the same, viz., to cut down and remove the necrosed bone and the impacted tooth, but it is not necessary to enter further into detail here.

